# Prospective association between sleep-related factors and the trajectories of cognitive performance in the elderly Chinese population across a 5-year period cohort study

**DOI:** 10.1371/journal.pone.0222192

**Published:** 2019-09-06

**Authors:** Tingting Sha, Wenwei Cheng, Yan Yan

**Affiliations:** 1 Department of Epidemiology and Medical Statistics, Xiangya School of Public Health, Central South University, Changsha, Hunan, China; 2 Third Xiangya Hospital of Central South University, Changsha, Hunan, China; Istituto Di Ricerche Farmacologiche Mario Negri, ITALY

## Abstract

The integral role of sleep in cognition, such as night-time sleep and napping duration, has yielded mixed findings, especially in healthy elderly adults. This study aimed to identify the heterogeneous classes of the cognitive trajectories and investigated the associations between sleep parameters and the trajectories of cognition in different elderly subpopulations. The study was based on a large, national representative sample aged 60 years or older. Two cognitive measures were assessed, including executive function and episodic memory. Sleep parameters were evaluated, including post-lunch napping, night-time sleep duration, and sleep disturbances. Latent growth mixture model (LGMM) was used to describe the trajectories of cognition and investigate the effects of sleep factors on cognition. Three heterogeneous trajectories were identified for executive cognition and four for episodic memory. Inverted U-shape associations of cognition with night-time sleep and napping duration were found. In LGMM, night-time sleep duration was negatively associated with the baseline episodic memory in elderly adults. Post-lunch napping was positively associated with the baseline executive function (*β* = 0.078, *P*<0.05) and episodic memory (*β* = 0.084, *P*<0.05) in men, whereas it was only associated with impaired episodic memory (*β* = -0.152, *P*<0.05) in women. Frequent sleep disturbances were only associated with the impaired executive function at baseline (*β* = -0.088, 95%CI -0.162, -0.013) among older men. Overall, sleep parameters played different roles in heterogeneous trajectories of cognition by sex difference. Sleep factors may not be related to the rate of cognition decline, but these factors, independent of time-variant depressive symptoms, were associated with the initial status of cognition at baseline.

## Introduction

By the year 2050, the population aged 60 years and older is expected to reach more than 2 billion, accounting for almost 20% of the whole population [1]. Cognitive impairment is known to be associated with increased risks of disability, physical limitations, and reducing the quality of life in elderly adults [2], and has become a significant public health issue worldwide [3]. As one of the most rapidly aging societies, China has 7.4 million elderly people currently living with dementia [4]. Given its enormous socioeconomic burden, it is imperative to identify additional modifiable lifestyle factors to decrease the incidence of cognitive impairment.

Sleep problem has been considered to be a modifiable behavioral factor adversely affecting public health [5–7]. As age growth, sleep patterns changed significantly with increased awakenings and decreased night-time sleep duration [8]. In China, approximately 40% of adults aged 60 years or over experienced sleep complaints [9]. Over the last two decades, there has been a growing awareness of the integral role that sleep may affect cognitive performance. However, its role in cognition has yielded mixed findings, especially in healthy elderly adults [10].

Prospective non-demented elderly cohort studies found that lower sleep efficiency and greater nighttime wakefulness were related to the risk of subsequent cognition decline [11, 12], or Alzheimer’s disease [13]. Early evidence showed that self-reported sleep duration was contributed to the cognitive impairment [14–16], but findings from other studies showed no association between self-reported insomnia, sleep duration and cognitive decline in elderly [17–19]. Previous studies suggest that daytime napping in older adults may compensate for insufficient nighttime sleep and potentially enhance cognitive performance [20–22]. However, other studies indicated that longer daytime naps might contribute to both a sedentary lifestyle and impaired social engagement, both of which are associated with cognitive impairment [6, 23, 24].

Several gaps challenge the existed results. First, most prior studies regarding sleep and cognition in older adults focused on nighttime sleep rather than daytime napping [6, 19, 25]. Moreover, most studies categorized the study sample into two groups, such as nappers and non-nap-pers, rather than any level of napping duration [6, 26]. Second, older women always have more frequent sleep problems and shorter sleep duration than men [27, 28]. However, few studies investigate the sex difference in the association between sleep and cognition. Additionally, depressive symptomatology might moderate the association of sleep quality with cognitive function among the elderly [29, 30], whereas most previous studies only adjusted for the depressive symptoms at baseline and the lasting effect of depressive symptoms on this association has scarcely been investigated. Lastly, most prior studies established their models based on the means of the entire population, while the trajectory of the cognition is not the same for all old adults. Research estimating different classes of cognition growth trajectories over time is rare, which will mask group differences in cognitive performance trajectories.

Sleep difficulties could particularly be salient issues among older adults[8, 31], thus, understanding the role of sleep on cognition are of immense interest for clinical research, specifically in those over the age of 60 years who are inclined to have a higher risk of developing cognitive impairment or dementia. In this study, we tried to bridge the gaps in the existing literature and aimed to address two following questions using the latent growth mixture model (LGMM) in a large, population-based sample derived from China Health and Retirement Longitudinal Study (CHARLS): (1) To describe the trajectories of two cognitive functions—executive function and episodic memory for different Chinese subpopulations. (2) To investigate the association of nighttime sleep, post-lunch napping duration, and sleep disturbances with cognition by gender difference.

## Methods

### Data and sample

This study was based on the national data from CHARLS, a population-based longitudinal survey conducted by the National School of Development of Peking University from 2011 to 2016. A multistage probability sampling design and a probability proportional sampling technique were adopted in the baseline survey to ensure the representation of the sample. Detailed of the sampling procedure and the description of the CHARLS are available in a previous study [32]. Briefly, the residents aged 45 years or older living in China (including 28 provinces, except Tibet) were first interviewed through a face-to-face computer-assisted personal interview in June 2011 and were followed up every 2 years. The national baseline survey was conducted with 17,708 individual participants in 2011–2012, and 15,331 participants completed the three follow-ups. After excluding the sample with missing data of anthropometric and physical performance measures (n = 1129), cognitive performance (n = 2960), sleep measures (n = 495), depression measures (n = 828), as well as excluding the participants who were less than 60 years old (n = 6254) and had psychiatric problems, memory-related diseases, brain damage and stroke (n = 81), a subset of 3584 older adults were included in the final analysis, with age ranging from 60 to 98 years old.

### Measurement of cognitive performance

Based on the American Health and Retirement Study, two cognition measures were used in the CHARLS research [33]. The first measure was the executive function, which was evaluated through the Telephone Interview of Cognitive Status (TICS) battery as well as figure drawing test. TICS was a reliable and valid method as Mini-Mental State Examination used to screen cognitively impaired elderly [34], involving ten following questions, including recalling the today’s date (month, day, year), the day of week and season of the year, serial 7 subtraction from 100 (up to five times). Figure drawing test is to ask the participants to repaint a picture presented to him/her. Participants who successfully redraw the picture received a score of 1 and those who failed received a score of 0. All of the answers to these questions are aggregated into a participant’s executive function score, ranging from 0 to 11. The second cognition measure was the episodic memory through immediate and delayed recall. After the interviewers reading a list of ten unrelated Chinese words, the participants are asked to repeat the words in any order immediately (immediate word recall). About four minutes later, the respondents were asked to recall the list of the words again (delayed recall). Episodic memory was calculated as the mean of immediate and delayed recall scores based on the method used by Lei et al. [33]. The total score of episodic memory ranged from 0 to 10.

### Measurement of sleep measurements

Similar to the Pittsburgh Sleep Quality Index [35], self-reported nighttime sleep duration and sleep disturbance were assessed in this study. Nighttime sleep duration was assessed by asking: “During the past month, how many hours of actual sleep did you get at night (average hours for one night)? (This may be shorter than the number of hours you spend in bed).” We categorized the respondents into four nighttime sleep groups [36, 37]: short sleeper (<5 hours), somewhat short sleeper (5–7 hours), normal sleeper (7–9 hours), and long sleeper (≥9 hours). Sleep disturbance was assessed using the statement to report the frequency of “My sleep was restless”. According to how he/she felt and behaved during the last week, with answers varying from “rarely or none of the time (<1 day)”, “some of the time (1–2 days)”, “much or a moderate amount of the time (3–4 days)”, and “most or all the time (5–7 days)”. Additionally, we also collected the information on the post-lunch napping duration by asking: “During the past month, how long did you take a nap after lunch in general?” Based on the early evidence [38, 39], respondents were classified into four groups, non-nappers (0 minutes), short nappers (<30 minutes), moderate nappers (30–90 minutes), and long nappers (>90 minutes).

### Measurement of other covariates

Based on the previous literature, potential confounders at baseline were adjusted, including demographic characteristics, lifestyle and health behaviors, and doctor diagnoses of chronic diseases. Demographic characteristics included age, gender, education level (illiterate, Primary school or below, High/vocational school, and College or above), marital status (current married, separated, divorced, widowed, and never married), hukou status (agricultural hukou and non-agricultural hukou), weight, height, and body mass index. Hukou status was the registration system in China created in 1955 to restrict internal population movement, divided Chinese into 2 categories: agricultural hukou and non-agricultural hukou.

Lifestyle and health behaviors were included in cigarette smoking and alcohol drinking, depressive symptoms, and activities of daily living (ADL). In CHARLS, smoking was defined as “current smokers” and “non-current smokers”. Drinking status was defined as ever drank alcoholic beverages in the year, and participants were classified into “never drinkers”, “current drinkers”, or “former drinkers”. Depressive symptoms were evaluated at each time point using the 10-item Center for Epidemiologic Studies Depression Scale short form [40]. Each item was scored on a 4-point Likert scale, with a total number ranging from 0 to 30. The activities of the daily living (ADL) were assessed at three follow-ups using five types of instrumental activities of daily living and six types of daily activities, with answers varying from “no difficulty” to “much difficulty”, ranging from 0 to 3.

Doctor diagnoses of chronic diseases were collected through the respondents’ self-report, including hypertension, diabetes, or high blood sugar, heart problems, dyslipidemia, and other chronic diseases. The number of self-reported health comorbid diseases were coded into three groups (0, 1–2, and ≥ 3) at the baseline survey.

### Data analysis

Continuous variables were presented as mean (standard deviation), if distributed normally, and categorical variables were presented as numbers and proportions. Continuous variables were compared using two-sample t-tests or Wilcoxon’s signed-rank tests. Chi-square tests or Fisher’s exact tests were used to compare the categorical outcomes. The one-way ANOVA analysis was used to compare the cognition differences within distinct sleep problems by gender group.

To model the heterogeneity in the subpopulations or subclasses, we employed the LGMM method to identify distinct trajectories of cognition. Unlike the traditional latent growth curve models, which based on the assumption that the data were drawn from a single homogenous observed population [41], LGMM was a method for identifying multiple unobserved subpopulations, describing longitudinal change with each unobserved sub-population, and examining differences in change among unobserved subpopulations [42]. The trajectory of cognition over time was modeled with two continuous latent variables, latent intercept growth factor, representing the initial status of the cognitive performance, and latent slope growth factor, reflecting the rate of the cognition changes. The different subpopulations were modeled using the categorical latent variables (classes) in LGMM.

Two steps of the data analysis in this study were employed to identify the latent classes of two cognition trajectories and test the predictors of the latent trajectories by gender differences. Because of the three times of follow-up, the heterogeneous classes of the cognitive trajectories were modeled with specified linear LGMM in this study. The first step of the model specification was to identify the appropriate number of classes that yielded the most desirable fit. We compared one- to six-class unconditional LGMM models (with no covariates or predictors). The best-fitting LGMM of the cognitive performance trajectories was with the lowest information criteria, high entropy for the confidence of classification [42]. Additionally, the output results were carefully reviewed for any cautionary notes generated by Mplus, such as non-convergence, and small sample sizes. In the second stage, we extended the unconditional LGMM to conditional LGMM with primary sleep factors and the controlling covariates. Finally, in post hoc analyses, we tested the effect of predictors on the identified latent classes of the cognitive performance changes.

A variety of model-fit indices were used to evaluate the goodness of LGMM: χ2 statistic, likelihood ratio chi-square, Akaike Information Criterion (AIC), Bayesian Information Criterion (BIC), and sample size–adjusted BIC and entropy. A higher value indicated less classification error, and the model had adequate classification quality if the value was >0.8 [43]. Lo-Mendell-Rubin adjusted likelihood ratio test (LMR-test) was used to compare n-class model versus n−1 class models. The significant p-value suggested that the n-class model was well improved over the n-1-class model. The models were estimated using the robust maximum likelihood approach. One hypothesis in this research was that data were missing at random. Missing data were modeled using a full-information maximum-likelihood method which estimates the parameters based on all available data and provides the robust estimates in the presence of non-normality and non-independence of observations [44, 45].

The descriptive statistics were conducted using SAS version 9.4 (SAS Institute). LGMM analyses were performed using Mplus, version 7.0 (Muthén & Muthén). Significance in all tests was based on a two-tailed P<0.05.

### Ethics approval and consent to participate

Each participant included in this study signed a written informed consent form before taking the survey. Ethics approval for the data collection in CHARLS was obtained from the Biomedical Ethics Review Committee of Peking University (IRB00001052-11015). Ethics approval for the use of CHARLS data was obtained from the University of Newcastle Human Research Ethics Committee (H-2015-0290).

## Results

### Descriptive analysis

A total of 3584 individuals were included in this study, involving 1893 men and 1691 women. The baseline characteristics of the participants are summarized in Table 1. The mean age of the overall population, men and women, were 66.59±5.50 years, 66.70±5.50 years, and 66.39±5.50 years, respectively. The differences in sample characteristics between elderly males and females in China are also presented in Table 1. There were significant differences (P<0.05) between males and females with respect to age, BMI, marital status, education, Hukou, smoking, drinking, number of comorbid diseases, depression, ADLs, nighttime sleep duration, post-lunch napping duration, sleep disturbance, episodic memory, and executive function. Compared to elderly men, women were more likely to have sleep problems and worse cognitive performance.

**Table 1:** Basic characteristics of the participants for the overall sample.

Variables	Overall N = 3584	Men N = 1893	Women N = 1691	*P*
Age (years), mean±SD	66.59±5.50	66.7±5.50	66.3±5.50	0.039
BMI (kg/m^2^), mean±SD	22.96±3.67	22.46±3.45	23.53±3.92	<0.001
Marital status, %				
Married	82.8	88.6	76.5	<0.001
Separated	0.6	0.6	0.6	
Divorced	0.5	0.7	0.3	
Widowed	15.2	8.6	22.6	
Never married	0.8	1.5	0	
Educational level, %				
Illiterate	30.7	15.8	47.5	<0.001
Primary school or below	50.3	58.7	41.0	
High/vocational school	17.5	23.5	10.8	
College or above	1.4	2.0	0.8	
Hukou status, %				
Agricultual Hukou	80.3	78.1	82.7	<0.001
Non-agricultural Hukou	19.7	21.9	17.3	
Cigarette smoking, %				
Current smokers	43.6	73.4	10.3	<0.001
Non-current smokers	56.4	26.6	89.7	
Alcoholic drinking, %				
Current drinkers	25.9	41.9	8.0	<0.001
Former drinkers	6.4	8.8	3.7	
Ever drinkers	67.6	49.2	88.2	
Number of chronic diseases, %				
0	60.7	64.4	56.5	<0.001
1–2	35.7	24.3	27.4	
≥3	3.0	11.3	16.1	
Nighttime sleep duration (hours), %				
<5	19.2	15.2	23.7	<0.001
5–7	35.4	36.9	33.6	
7–9	37.5	39.5	35.3	
≥9	8.0	8.4	7.5	
Postlunch napping duration (minutes), %				
0	44.8	38.9	51.4	<0.001
<30	9.3	7.5	11.4	
30–90	31.3	35.7	26.4	
≥90	14.5	17.9	10.8	
Sleep disturbance (days), %				
<1	49.2	57.0	40.4	<0.001
1–2	16.8	15.9	17.9	
3–4	15.4	11.3	20.0	
5–7	18.6	15.8	21.7	
ADL in 2011, mean±SD	12.09±2.51	11.83±2.15	12.39±2.83	<0.001
ADL in 2013, mean±SD	12.24±2.80	12.02±2.65	12.48±2.94	<0.001
ADL in 2015, mean±SD	12.76±3.78	12.45±3.55	13.12±4.00	<0.001
Depressive symptom in 2011, mean±SD	8.56±6.20	7.65±5.79	9.58±6.48	<0.001
Depressive symptom in 2013, mean±SD	8.00±5.75	7.17±5.28	8.92±6.10	<0.001
Depressive symptom in 2015, mean±SD	8.49±6.54	7.46±6.02	9.64±6.90	<0.001
Executive function in 2011, mean±SD	7.01±3.07	7.88±2.75	6.04±3.10	<0.001
Executive function in 2013, mean±SD	6.81±3.20	7.69±2.89	5.83±3.24	<0.001
Executive function in 2015, mean±SD	6.50±3.22	7.38±2.88	5.53±3.31	<0.001
Episodic memory in 2011, mean±SD	3.01±1.74	3.14±1.70	2.94±1.79	0.001
Episodic memory in 2013, mean±SD	3.12±1.72	3.27±1.69	2.96±1.73	<0.001
Episodic memory in 2015, mean±SD	2.69±1.77	2.79±1.77	2.58±1.76	<0.001

Abbreviation: ADL, Activities of daily living; SD, standard deviation.

Changes in executive function according to different sleep factors are presented in Table 2. The elderly males exhibited better cognitive performance than females over time. The changes of executive function for Chinese elderly showed an inverted ‘U’ shaped association between different nighttime sleep duration and nap duration, indicated that moderate napping or nighttime sleep duration were associated with higher cognition scores. Sleep disturbances were associated with impaired cognitive performance. Similar results had also been found in episodic memory, which can be acquired in S1 Table.

**Table 2:** Levels of executive function according to different sleep-related factors among the Chinese elderly.

Variable	No.	2011		2013		2015	
		mean±SD	*P*	mean±SD	*P*	mean±SD	*P*
**Male**							
Nighttime sleep duration (hours)							
<5	287	7.15±2.86	<0.001	7.04±3.00	<0.001	6.49±3.06	<0.001
5–7	699	8.10±2.68		7.86±2.77		7.72±2.74	
7–9	748	8.11±2.69		7.98±2.80		7.55±2.77	
≥9	159	7.54±2.79		7.15±2.98		6.77±3.03	
Post-lunch napping duration (minutes)							
0	737	7.66±2.84	0.005	7.47±2.95	0.001	7.19±2.90	0.040
<30	142	8.08±2.59		7.70±2.81		7.46±3.00	
30–90	676	8.18±2.73		8.08±2.81		7.62±2.78	
≥90	338	7.89±2.55		7.58±2.72		7.33±2.88	
Sleep disturbance (days)							
0	1079	8.08±2.71	0.001	7.84±2.88	0.039	7.50±2.85	0.054
1–2	301	8.02±2.61		7.84±2.59		7.50±2.73	
3–4	213	7.52±2.92		7.45±3.03		7.01±3.07	
5–7	300	7.51±2.80		7.38±2.88		7.16±2.87	
**Female**							
Nighttime sleep duration (hours)							
<5	400	5.40±3.07	<0.001	5.12±3.18	<0.001	4.92±3.29	<0.001
5–7	568	6.36±3.04		6.25±3.24		5.85±3.24	
7–9	697	6.31±3.10		6.05±3.26		5.75±3.33	
≥9	126	5.39±3.10		5.13±2.89		4.98±3.24	
Postlunch napping duration (minutes)							
0	870	5.81±3.13	0.003	5.56±3.26	<0.001	5.23±3.28	<0.001
<30	193	6.64±2.87		7.08±3.24		6.33±3.43	
30–90	446	6.25±3.14		5.90±3.18		5.75±3.34	
≥90	182	6.02±3.04		5.59±3.04		5.57±3.02	
Sleep disturbances (days)							
0	684	6.30±3.04	<0.001	5.86±3.24	0.017	5.72±3.29	0.036
1–2	302	6.37±3.26		6.22±3.19		5.68±3.28	
3–4	338	5.73±3.03		5.86±3.23		5.44±3.27	
5–7	367	5.59±3.09		5.43±3.28		5.13±3.35	

Abbreviation: SD, standard deviation.

### Unconditional LGMM

In order to determine the best-fitting model for cognition, we established six different unconditioned LGMMs incrementally, varied from one-class model to the six-class model. The possible six trajectories models for two cognitive measures by sex are presented in Table 3. After compared the fitness statistics comprehensively, the three-class model was identified as the optimal fitting model representing executive function changes for older men and women, with the highest entropy (0.850 and 0.781). The P-value (P<0.001) of LRT-test showed a significant difference between the 2-class and 3-class model, indicated a better fitting of the 3-class model. Additionally, the results of LRT-test of the 4-class or 5-class models showed both non-significant P-values, indicated that the 4-class or 5-class model failed to provide a better improvement of the goodness-fit-indices. The results of the optimal fitting model for executive function in women were similar to those in men. Similarly, in combination with these indices, the 4-class model was identified as the appropriate class solution to represent the episodic memory for elderly Chinese.

**Table 3:** Fit indices for one- to six-class growth mixture models for executive function and episodic memory among Chinese elderly.

	LL-value	AIC value	BIC value	Adjusted-BIC value	LRT-test	Entropy
**Male**						
Executive function						
1-class	-13241.24	26498.47	26542.84	26517.42	-	-
2-class	-13107.54	26237.08	26298.08	26263.14	<0.001	0.760
3-class	-12998.44	26024.88	26102.53	26058.05	<0.001	0.850
4-class	-12971.98	25977.97	26072.25	26018.24	0.056	0.795
5-class	-12932.91	25905.82	26016.74	25953.20	0.276	0.832
6-class	-12889.63	25825.25	25952.81	25879.74	0.117	0.835
Episodic memory						
1-class	-10798.57	21613.15	21657.52	21632.10	-	-
2-class	-10778.21	21578.42	21639.43	21604.48	<0.001	0.495
3-class	-10767.86	21563.72	21641.36	21596.89	0.419	0.616
4-class	-10758.51	21551.03	21645.31	21591.30	0.011	0.666
5-class	-10751.68	21543.37	21654.29	21590.75	0.078	0.678
6-class	-10740.18	21526.36	21653.92	21580.85	0.059	0.619
**Female**						
Executive function						
1-class	-12033.46	24082.91	24126.37	24100.96	-	-
2-class	-11949.51	23921.02	23980.78	23945.84	0.004	0.690
3-class	-11907.25	23842.50	23918.56	23874.09	<0.001	0.781
4-class	-11877.99	23789.97	23882.34	23828.33	<0.001	0.699
5-class	-11839.46	23718.92	23827.58	23764.05	0.013	0.733
6-class	-11820.35	23686.70	23811.66	23738.59	0.026	0.763
Episodic memory						
1-class	-9752.40	19520.80	19564.26	19538.85	-	-
2-class	-9736.48	19494.96	19554.72	19519.78	<0.001	0.664
3-class	-9717.87	19463.75	19539.80	19495.33	0.101	0.667
4-class	-9703.49	19440.98	19533.34	19479.33	0.094	0.714
5-class	-9698.19	19436.37	19545.03	19481.49	0.310	0.643
6-class	-9687.43	19420.86	19545.81	19472.74	0.551	0.671

Abbreviation: AIC, Akaike Information Criterion; BIC, Bayesian Information Criterion; LL, Likelihood; LMR-test, Lo-Mendell-Rubin adjusted likelihood ratio test.

### Conditional LGMM

**Fig 1:**
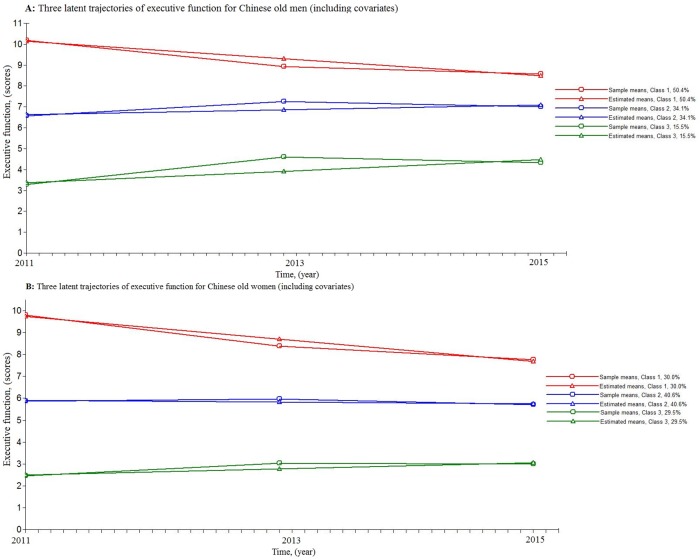
Three latent trajectories of executive function for elderly adults (including covariates). (A) Panel A displays three latent trajectories of executive function for elderly men (including covariates). (B) Panel B displays three latent trajectories of executive function for elderly women (including covariates).

Based on the optimal unconditional fitting model for cognition, we controlled for the sleep-related variables, time-variant covariates, such as depressive symptoms and ADL, as well as demographic characteristics, including age, education, marital status, Hukou, smoking, drinking and number of comorbid diseases and established the conditional LGMMs. Class assignment and intercept growth parameter were regressed on all of the covariates. Table 4 shows intercept and growth parameter estimates for the latent conditional trajectories of executive function and episodic memory by gender. The distinct latent classes for the executive function between the aged men and women are shown in Fig 1. For the executive function among elderly men, more than half of sample (50.4%) was categorized into class 1 (sharped cognition decline) with highest initial levels of cognitive scores and a subsequent significant decline of the cognition across time. Approximately a third of participants were estimated to be classified into class 2 (stable), with a moderate baseline executive function and a stable cognition change. Class 3 (slight cognition increase) was characterized by low initial executive function and a slight increase in cognition across time. The results in executive function among elderly women were similar to those in elderly men, but the proportion of class 1 (30.0%) attenuated while class 2 (40.6%) and class 3 (29.5%) increased. Similarly, the latent classes of episodic memory were shown in S1 Fig.

**Table 4:** Growth factor parameter estimates for LGMM model for cognition by gender.

Class	Intercept		Slope	
	*β*(95%CI)	SE	*β*(95%CI)	SE
**Male**				
Executive function				
Class 1	10.13 (10.04 to 10.23)***	0.05	-0.83 (-0.91 to -0.74)***	0.04
Class 2	6.62 (6.45 to 6.79)***	0.09	0.23 (0.11 to 0.36)***	0.06
Class 3	3.36 (3.10 to 3.61)***	0.13	0.56 (0.33 to 0.79)***	0.12
Episodic memory				
Class 1	4.76 (4.31 to 5.21)***	0.23	0.39 (0.14 to 0.65)***	0.13
Class 2	2.97 (2.76 to 3.18)***	0.11	-0.78 (-0.88 to -0.67)***	0.05
Class 3	3.66 (3.50 to 3.83)***	0.09	0.04 (-0.08to 0.15)	0.07
Class 4	1.90 (1.28 to 2.53)***	0.31	1.91 (1.29 to 2.53)***	0.32
**Female**				
Executive function				
Class 1	9.74 (9.58 to 9.87)***	0.08	-1.03 (-1.16 to -0.91)***	0.07
Class 2	2.50 (2.31 to 2.68)***	0.09	0.28 (0.16 to 0.40)***	0.06
Class 3	5.91 (5.72 to 6.09)***	0.09	-0.08 (-0.19 to 0.04)	0.06
Episodic memory				
Class 1	2.70 (2.53 to 2.88)***	0.09	-0.87 (-1.00 to -0.75)***	0.08
Class 2	1.85 (-1.09 to 4.79)	1.50	3.29 (1.65 to 4.93)***	0.84
Class 3	3.59 (3.20 to 3.51)***	0.08	-0.15 (-0.28 to -0.03)***	0.06
Class 4	3.81 (3.48 to 4.14)***	0.17	0.62 (0.46 to 0.78)***	0.08

*P<0.05,

**P<0.01,

***P<0.001.

Table 5 shows the estimates of sleep parameters in the conditional model. After adjusting for covariates, no significant association was found between night-time sleep duration and executive function in elderly adults. Post-lunch sleep duration was significantly associated with baseline executive function for elderly men (*β* = 0.078, 95%CI 0.008, 0.148), but not for women (*β* = -0.039, 95%CI -0.150 to 0.073). Self-reported sleep disturbances were only related to impaired executive function at baseline for elderly men, suggesting that more frequency of sleep restless was associated with the impaired executive function (*β* = -0.088, 95%CI -0.162, -0.013). Longer nighttime sleep duration was associated with the poor episodic memory at baseline among elderly men and women (*β* = -0.075, 95%CI -0.174, -0.020 and *β* = -0.114, 95%CI -0.217, -0.010). Post-lunch napping duration played a different role in episodic memory for men and women. For elderly male, longer post-lunch napping duration was positively associated with episodic memory (*β* = 0.084, 95%CI 0.018, 0.149), while for elderly female, it was negatively associated with the episodic memory (β = -0.152, 95%CI -0.225, -0.079). There was no significant relationship of sleep disturbances with episodic memory among elderly adults.

**Table 5:** Estimates of covariate prediction of intercept and slope of the trajectories of different domains of cognition by gender.

Covariates	Sleep measurements, *β* (95%CI)		
	Night-time sleep duration	Post-lunch napping	Sleep disturbances
**Male**			
Executive function			
Intercept	0.015 (-0.079 to 0.109)	0.078 (0.008 to 0.148)*	-0.088(-0.162 to -0.013) *
Slope	-0.022(-0.0.93 to 0.050)	-0.009 (-0.059 to 0.040)	-0.014(-0.068 to 0.041)
Episodic memory			
Intercept	-0.075(-0.174 to -0.020)*	0.084(0.018 to 0.149) *	0.001(-0.071 to 0.072)
Slope	-0.026 (-0.090 to 0.038)	-0.027 (-0.070 to 0.016)	-0.018 (-0.064 to 0.028)
**Female**			
Executive function			
Intercept	0.003 (-0.150 to 0.157)	-0.039 (-0.150 to 0.073)	0.085 (-0.048 to 0.218)
Slope	0.046(-0.040 to 0.132)	0.013 (-0.050 to 0.077)	0.020 (-0.056 to 0.096)
Episodic memory			
Intercept	-0.114 (-0.217 to -0.010) *	-0.152 (-0.225 to -0.079) *	0.056 (-0.047 to 0.135)
Slope	0.023 (-0.042 to 0.089)	0.040 (-0.007 to 0.087)	0.014 (-0.037 to 0.064)

Abbreviation: 95%CI, 95% confidence interval.

*P<0.05.

Comparisons of the sleep-related factors on the 3-class trajectories of executive function based on the post hoc logistic regression are presented in Table 6. For Chinese men, the class 1 comprised the largest number of elderly men and was the main focus of our concern, we selected the class 1 as the referent class and used the logistic regressions to investigate the associations of different sleep factors with the different trajectories. When compared to class 1, participants who reported having 5 to 7 days sleep restlessly per week were associated with an increased probability of membership in class 2 (OR = 1.53, 95%CI 1.12, 2.10). In comparison to the class 1, participants who slept less than 9 hours were associated with a decreased probability of membership in class 3 (OR = 0.54, 95%CI 0.36, 0.80 and OR = 0.61, 95%CI 0.40, 0.93), while participants who reported having poor sleep quality more than 3 days a week were at increased probability of classifying into class 3 (OR = 1.76, 95%CI = 1.15–2.68, and OR = 1.57, 95%CI = 1.05–2.34). For older women, when compared to the referent class 1, long (≥90 minutes) and moderate nappers (30–90 minutes) were associated with increased probability of membership class 2 (OR = 1.32, 95%CI 1.00, 1.74 and OR = 1.64, 95%CI 1.11, 2.40). Participants who reported having a poor sleep quality more than 5 days a week were associated with a decreased probability of membership in class 2 (OR = 0.68, 95%CI 0.47, 0.97). In comparison to the referent class, participants who were short nappers (0–30 minutes) and moderate nappers (30–90 minutes) were at the increased probability of classifying into class 3 (OR = 2.11, 95%CI 1.42, 3.13 and OR = 1.48, 95%CI 1.10, 2.00). Similarly, findings from the S2 Table showed that each of the sleep-related factors played different roles in episodic memory among distinct latent classes for men and women.

**Table 6:** Covariate prediction of the trajectories of executive function based on the post hoc logistic regression.

Variables	OR	95%CI	OR	95%CI
**Male**		**Class 2**		**Class 3**
Nighttime sleep duration (hours)				
5–7	0.77	0.55–1.06	0.54*	0.36–0.80
7–9	0.80	0.57–1.12	0.61*	0.40–0.93
≥9	0.85	0.53–1.34	0.97	0.56–1.67
Post-lunch sleep duration (minutes)				
0–30	1.03	0.69–1.53	0.76	0.44–1.33
30–90	0.89	0.70–1.13	0.76	0.55–1.01
≥90	1.33	0.99–1.77	0.99	0.68–1.46
Sleep disturbances (days)				
1–2	1.21	0.91–1.61	0.96	0.64–1.45
3–4	1.28	0.90–1.81	1.76*	1.15–2.68
5–7	1.53*	1.12–2.10	1.57*	1.05–2.34
**Female**		Class 2		Class 3
Nighttime sleep duration (hours)				
5–7	1.33	0.96–1.86	1.28	0.89–1.84
7–9	1.08	0.76–1.53	1.05	0.71–1.56
≥9	1.04	0.63–1.72	0.59	0.32–1.08
Post-lunch sleep duration (minutes)				
0–30	1.24	0.83–1.85	2.11*	1.42–3.13
30–90	1.32*	1.01–1.74	1.48*	1.10–2.00
≥90	1.64*	1.11–2.40	1.19	0.76–1.86
Sleep disturbances (days)				
1–2	1.04	0.74–1.45	0.91	0.63–1.31
3–4	0.91	0.66–1.27	0.77	0.54–1.11
5–7	0.68*	0.47–0.97	0.69	0.46–1.02

Abbreviation: *OR* Odds ratio, *95%CI* 95% confidence interval.

*P<0.05.

## Discussions

This study described the heterogeneous classes of cognitive trajectories based a longitudinal panel survey of 3584 community-dwelling Chinese aged 60 and older and investigated the effects night-time sleep time, post-lunch napping duration and sleep disturbances on the heterogeneous cognitive trajectories. Our findings identified 3 heterogeneous executive function trajectories of longitudinal changes over 5 years among healthy older Chinese: class 1 (sharp cognitive decline), class 2 (stable) and class 3 (slight cognitive increase), and 4 trajectories were identified for episodic memory. Based on the LGMM approach, this study found that post-lunch napping duration and sleep disturbances had different effects on latent heterogeneous trajectories of episode memory and executive function among the elderly men and women.

This study found that sleep problems are common in Chinese older population, especially for older women. In univariate analyses, sleep-related factors were significantly associated with two cognitive measures. However, these effects were attenuated as time-variant depressive symptoms adjusted. This is not surprising, as previous research suggested that older adults with depression were related to the increased risks of frequent sleep disruption and the decreased neuropsychological function [46–48]. Depressive symptoms might mediate at least some of the association between subjective sleep complaints and cognitive decline [24]. Our results showed that independent of time-variant depression, sleep measures were significantly contributed to the baseline cognition for the elderly. Moreover, unlike women, post-lunch napping and sleep disturbances were only associated with executive function in men, indicated significant sex differences in cognition and differences in sleep parameters are the one cause of this.

Mounting evidence points out that sleep duration might be predictive of cognition outcomes in older adults. Prospective studies showed both self-reported long [16, 49, 50] and short [15, 24, 50] sleep duration were contributed to the cognitive impairment or dementia. Previous studies found a U-shaped association between sleep duration and cognitive outcomes [16, 50]. Consistent with the previous research [16, 50], in univariates analyses, our data showed an inverted U-shape association between cognition and nighttime sleep duration, suggesting that both short and long sleep duration may result in the poor cognitive performance. When controlling for covariates, our results showed that long sleep duration was associated with the impaired episodic memory among Chinese elderly, whereas no association was found between executive function and nighttime sleep of different duration.

The previous cross-sectional study documented that individual who napped for long tended to have a more unsatisfactory performance on neuropsychological tasks [51]. Better than the cross-sectional design, based on a prospective study, our study found an inverted U-shape association between cognition and post-lunch napping duration in the univariate analyses, suggesting that naps of a moderate duration were associated with better cognition than too much napping or the absence of napping in elderly adults. These findings were consistent with the previous studies [14, 24]. We also investigated the role of napping in cognition among individuals with different latent classes, which is seldom conducted by previous studies and is more appropriate for the real world. Our findings suggested that post-lunch napping was associated with a higher risk of cognitive impairment in episodic memory and executive function among elderly men, whereas it was only associated with impaired episodic memory in elderly women.

Previous research suggested that longer napping duration was associated with subsequent cognitive decline [24]. However, most studies were based on a hypothesis that the sample included in their studies were heterogeneous in the makeup of cognitive decline, as well as depression, history of depression and other variables, which might challenge their results [51]. Currently, we identified the latent classes of executive function and episodic memory and found that sleep-related parameters played different roles in different latent trajectories of cognition. Understanding trajectories of cognition are particularly important, which is beneficial to discover the individuals who are susceptible to poor cognition and rapid cognition decline as well as make the prevention more targeted.

Although, after controlling for covariates, sleep parameters were not associated with the rate of change in cognition, these factors were indeed associated with two cognitive measures at baseline. Our findings highlight the importance of public health implications that moderate post-lunch napping duration and night-time sleep time, fewer sleep disturbances were associated with better cognitive performance at baseline and may be beneficial to decrease the incidence of cognitive impairment in older general adults, specifically in those with amnestic mild cognitive impairment. This study has several strengths. CHARLES provides nationally representative panel data that enable inferences to be drawn about the Chinese elderly adults. Importantly, based on the LGMM approach, this study characterized the natural trajectories of cognitive performance for the non-demented Chinese elderly and examined the effects of sleep on different trajectories of cognition. Moreover, our study used three sleep parameters, instead of a single index, which can reflect an overall interact effect of the sleep factors, and provide more evidence on these association. Additionally, this study found a significant sex difference in the association of sleep with cognition. Last, besides assessing the baseline effect of the depression symptoms on the relationship between sleep and cognition, we also examined the effect of depressive symptoms over time and established a time-variant LGMM.

There are also several limitations to our study. First, 3-repeated measures of cognition in a 5-year period cohort made it impossible to scrutinize the nonlinear relationship between sleep and cognition, and we could only simply assume them in the linear association. Second, participants with missing data of critical variables were excluded in this study; this might introduce potential bias to our results. Then, sleep variables were measured by subjective self-report, not by objective actigraph measurement. Previous studies suggested actigraphy-assessed sleep was always shortened than self-reported sleep [52, 53]. Additionally, the assessment of cognition was mainly based on two domains of measurement. Thus, the ability to examine the degree of visuospatial deficits was limited, although this approach is similar to the Mini-Mental State Examination [54]. Last, although this study followed a 5-year period of observation, the suggested association must be interpreted cautiously because they were generated at a period that might not be long enough to discover the apparent cognitive decline. Hence, we will continue following up this study and test this association over a longer time period.

## Conclusions

In summary, different heterogeneous classes of the cognitive trajectories of two cognitive functions were identified in this cohort. Sleep is associated with the initial status of cognition and appear to be independent of time-variant depressive symptoms, while it is not related to the subsequent cognition decline among elderly adults. Further well-designed, population-based prospective studies with actigraphy-assessed sleep parameters, are needed to investigate the association between the changes in sleep and cognitive status.

## Supporting information

S1 Fig Four latent trajectories of episodic memory for elderly adults. (DOCX)

S1 Table Levels of episodic memory according to sleep-related factors among the elderly over time. (DOCX)

S2 Table Covariate prediction of trajectory for episodic memory based on the Post hoc logistic regression. (DOCX)
