# Supplementary material for: Prospective association between sleep-related factors and the trajectories of cognitive performance in the elderly Chinese population across a 5-year period cohort study
Source: PLoS One. 2019 Sep 6;14(9):e0222192. doi: 10.1371/journal.pone.0222192 (PMC6730942; doi:10.1371/journal.pone.0222192)
Supplement: S1 Fig — (DOCX) [file pone.0222192.s001.docx]

**
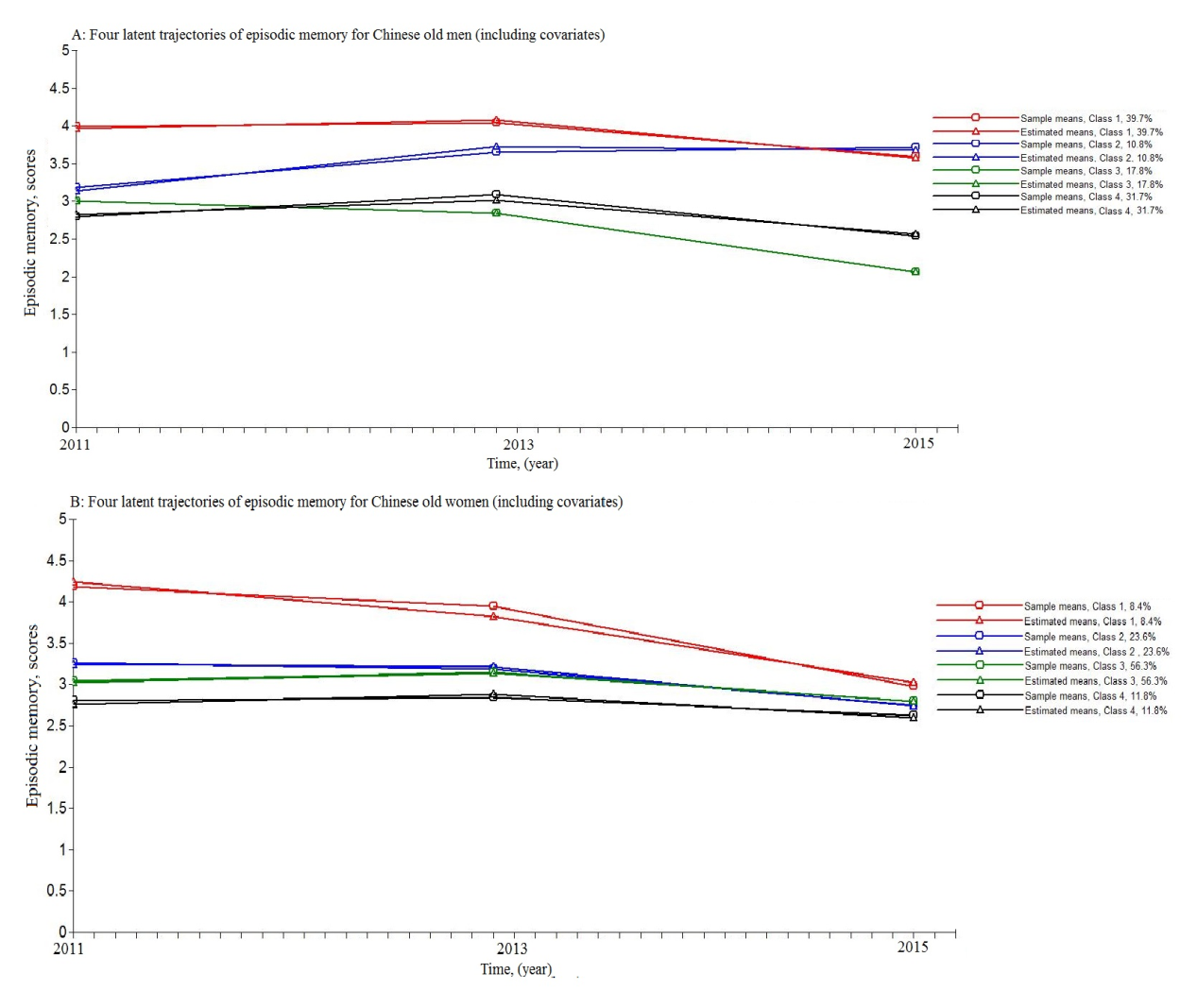
**

**S1 Fig. Four latent trajectories of episodic memory for elderly adults.** (A) Panel A displays four latent trajectories of episodic memory for elderly men (including covariates). (B) Panel B displays four latent trajectories of episodic memory for elderly women (including covariates).
