# Supplementary material for: Prospective association between sleep-related factors and the trajectories of cognitive performance in the elderly Chinese population across a 5-year period cohort study
Source: PLoS One. 2019 Sep 6;14(9):e0222192. doi: 10.1371/journal.pone.0222192 (PMC6730942; doi:10.1371/journal.pone.0222192)
Supplement: S1 Table — (DOCX) [file pone.0222192.s002.docx]

**S1 Table. Levels of episodic memory according to sleep-related factors among the elderly over time.**

| **Variable** | **no.** | **2011** | | | **2013** | | | **2015** | |
| --- | --- | --- | --- | --- | --- | --- | --- | --- | --- |
|  |  | **mean±SD** | **P** | **mean±SD** | | **P** | **mean±SD** | | **P** |
| **Male** |  |  |  |  | |  |  | |  |
| Nighttime sleep duration, hours | | |  |  | |  |  | |  |
| <5 | 287 | 2.83±1.63 | 0.008  (F=3.95) | 2.90±1.62 | | <0.001  (F=8.02) | 2.37±1.70 | | <0.001  (F=7.40) |
| 5-7 | 699 | 3.24±1.63 |  | 3.25±1.69 | |  | 2.88±1.77 | |  |
| 7-9 | 748 | 3.17±1.75 |  | 3.45±1.70 | |  | 2.89±1.77 | |  |
| ≥9 | 159 | 3.09±1.79 |  | 3.09±1.73 | |  | 2.64±1.70 | |  |
| Napping duration, minutes | | |  |  | |  |  | |  |
| 0 | 737 | 3.08±1.75 | 0.66  (F=0.53) | 3.26±1.69 | | 0.188  (F=1.60) | 2.66±1.74 | | 0.04  (F=2.77) |
| <30 | 142 | 3.24±1.61 |  | 3.26±1.75 | |  | 2.76±1.86 | |  |
| 30-90 | 676 | 3.17±1.69 |  | 3.35±1.72 | |  | 2.93±1.73 | |  |
| ≥90 | 338 | 3.15±1.62 |  | 3.11±1.61 | |  | 2.77±1.83 | |  |
| Sleep disturbances, days | | |  |  | |  |  | |  |
| 0 | 1079 | 3.24±1.73 | 0.003  (F=4.62) | 3.34±1.74 | | 0.046  (F=2.67) | 2.85±1.81 | | 0.043  (F=2.72) |
| 1-2 | 301 | 3.16±1.71 |  | 3.29±1.60 | |  | 2.86±1.79 | |  |
| 3-4 | 213 | 2.87±1.60 |  | 3.13±1.55 | |  | 2.55±1.54 | |  |
| 5-7 | 300 | 2.93±1.60 |  | 3.06±1.69 | |  | 2.64±1.70 | |  |
| **Female** |  |  |  |  | |  |  | |  |
| Nighttime sleep duration, hours | | |  |  | |  |  | |  |
| <5 | 400 | 2.64±1.79 | <0.001  (F=6.16) | 2.7±1.67 | | 0.003  (F=4.79) | 2.24±1.69 | | <0.001  (F=7.06) |
| 5-7 | 568 | 2.96±1.80 |  | 3.04±1.7 | |  | 2.70±1.76 | |  |
| 7-9 | 697 | 3.13±1.78 |  | 3.08±1.71 | |  | 2.71±1.76 | |  |
| ≥9 | 126 | 2.88±1.74 |  | 2.84±1.87 | |  | 2.48±1.86 | |  |
| Napping duration, minutes | | |  |  | |  |  | |  |
| 0 | 870 | 2.85±1.80 | 0.052  (F=2.58) | 2.86±1.76 | | 0.007  (F=4.07) | 2.47±1.77 | | 0.038  (F=2.82) |
| <30 | 193 | 3.24±1.79 |  | 3.33±1.62 | |  | 2.83±1.77 | |  |
| 30-90 | 446 | 2.98±1.77 |  | 2.99±1.71 | |  | 2.62±1.75 | |  |
| ≥90 | 182 | 2.96±1.78 |  | 2.97±1.69 | |  | 2.71±1.73 | |  |
| Sleep disturbances, days | | |  |  | |  |  | |  |
| <1 | 684 | 3.1±1.82 | <0.001  (F=8.93) | 3.02±1.79 | | 0.006  (F=4.19) | 2.64±1.77 | | 0.174  (F=1.66) |
| 1-2 | 302 | 3.19±1.83 |  | 3.20±1.78 | |  | 2.67±1.83 | |  |
| 3-4 | 338 | 2.74±1.67 |  | 2.81±1.65 | |  | 2.52±1.66 | |  |
| 5-7 | 367 | 2.63±1.76 |  | 2.80±1.63 | |  | 2.43±1.79 | |  |

Abbreviation: SD= standard deviation.
