# Supplementary material for: Prospective association between sleep-related factors and the trajectories of cognitive performance in the elderly Chinese population across a 5-year period cohort study
Source: PLoS One. 2019 Sep 6;14(9):e0222192. doi: 10.1371/journal.pone.0222192 (PMC6730942; doi:10.1371/journal.pone.0222192)
Supplement: S2 Table — (DOCX) [file pone.0222192.s003.docx]

**S2 Table. Covariate prediction of trajectory for episodic memory based on the Post hoc logistic regression**.

| **Variables** |  | |  |  | |  |  | |
| --- | --- | --- | --- | --- | --- | --- | --- | --- |
|  | **OR** | **95%CI** |  | **OR** | **95%CI** |  | **OR** | **95%CI** |
| **Male** |  | Class 1 |  |  | Class 2 |  |  | Class3 |
| Nighttime sleep duration (hours) | |  |  |  |  |  |  |  |
| <5 | 87.14^*^ | 43.80 -173.36 |  | 4.22^*^ | 2.06-8.62 |  | 2.03 | 0.92-4.49 |
| 5-7 | 5.72^*^ | 4.00-8.19 |  | 1.57^*^ | 1.12-2.18 |  | 1.19 | 0.82-1.72 |
| ≥9 | 0.16 | 0.06-0.42 |  | 0.60 | 0.37-0.97 |  | 1.33 | 0.83-2.13 |
| Post-lunch sleep duration (minutes) | |  |  |  |  |  |  |  |
| 0 | 0.66 | 0.37-1.18 |  | 1.12 | 0.68-1.86 |  | 0.35^*^ | 0.19-0.67 |
| 30-90 | 1.27 | 0.70-2.29 |  | 0.78 | 0.46-1.33 |  | 2.86^*^ | 1.58-5.16 |
| ≥90 | 2.17^*^ | 1.12-4.19 |  | 0.40 | 0.20-0.80 |  | 5.53^*^ | 2.92-10.48 |
| Sleep disturbances (days) |  |  |  |  |  |  |  |  |
| 1-2 | 0.54^*^ | 0.35-0.85 |  | 0.78 | 0.52-1.16 |  | 1.22 | 0.78-1.90 |
| 3-4 | 0.34^*^ | 0.20-0.57 |  | 0.50 | 0.30-0.83 |  | 1.31 | 0.77-2.23 |
| 5-7 | 0.15 ^*^ | 0.09-0.26 |  | 0.33 | 0.21-0.53 |  | 0.95 | 0.60-1.52 |
| **Female** |  | Class 1 |  |  | Class 2 |  |  | Class 4 |
| Nighttime sleep duration (hours) | |  |  |  |  |  |  |  |
| <5 | 0.12^*^ | 0.07-0.20 |  | 0.27^*^ | 0.12-0.58 |  | 0.15^*^ | 0.09-0.26 |
| 5-7 | 0.37^*^ | 0.27-0.51 |  | 0.47^*^ | 0.30-0.73 |  | 0.43^*^ | 0.29-0.65 |
| ≥9 | 4.57^*^ | 2.72-7.67 |  | 1.30 | 0.59-2.89 |  | 2.47^*^ | 1.16-5.25 |
| Post-lunch sleep duration (minutes) | |  |  |  |  |  |  |  |
| 0 | 1.24 | 0.76-2.00 |  | 1.02 | 0.56-1.85 |  | 2.07^*^ | 1.18-3.60 |
| 30-90 | 2.84^*^ | 1.73-4.67 |  | 1.24 | 0.65-2.37 |  | 0.92 | 0.48-1.77 |
| ≥90 | 4.92^*^ | 2.81-8.63 |  | 2.75 ^*^ | 1.32-5.76 |  | 0.29 | 0.08-1.07 |
| Sleep disturbances (days) |  |  |  |  |  |  |  |  |
| 1-2 | 1.06 | 0.74-1.52 |  | 0.36 | 0.21-0.61 |  | 1.10 | 0.63-1.92 |
| 3-4 | 0.96 | 0.66-1.41 |  | 0.11 | 0.05-0.26 |  | 2.73^*^ | 1.72-4.33 |
| 5-7 | 1.16 | 0.74-1.82 |  | 0.06 | 0.02-0.22 |  | 3.09^*^ | 1.84-5.20 |

Abbreviation: OR, Odds ratio; 95%CI, 95% confidence interval.

^*^*P*<0.05.
